# Fluorinated galactose analogues inhibit Streptococcus pneumoniae biofilm formation

**DOI:** 10.1099/acmi.0.001196.v4

**Published:** 2026-11-05

**Authors:** Daniella L. Spencer, Charlotte Fairchild, Jeremy S. Webb, Bruno Linclau, Saul N. Faust, Raymond N. Allan

**Affiliations:** 1 School of Pharmacy, Faculty of Health and Life Sciences, De Montfort University, Leicester, UK; 2 School of Biological Sciences, University of Southampton, Southampton, UK; 3 National Biofilms Innovation Centre, University of Southampton, Southampton, UK; 4 NIHR Southampton Clinical Research Facility and Biomedical Research Centre, University Hospital Southampton NHS Foundation Trust, Southampton, UK; 5 Faculty of Medicines and Institute for Life Sciences, University of Southampton, Southampton, UK; 6 School of Chemistry and Chemical Engineering, University of Southampton, Southampton, UK; 7 Department of Organic and Macromolecular Chemistry, Ghent University, Krijgslaan 281-S4, 9000 Ghent, Belgium

**Keywords:** biofilm, galactose, metabolism, *Streptococcus pneumoniae*

## Abstract

*Streptococcus pneumoniae* is a major cause of morbidity and mortality worldwide, causing invasive diseases such as pneumonia, sepsis and meningitis, but also less severe localized infections like otitis media. To enable pneumococcal survival in the nutritionally challenging nasopharyngeal niche, it possesses an extensive repertoire of genes dedicated to the utilization of a wide range of carbohydrates. Galactose, the predominant carbohydrate found in the respiratory tract, is important in pneumococcal colonization and virulence and therefore represents an attractive target for the development of new treatments. In this preliminary study, we investigated the potential of fluorinated galactose analogues as a strategy for preventing and treating pneumococcal biofilms. The analogues were tested against planktonic pneumococci, and their effects on *in vitro* biofilms were assessed through biomass and viability measurements with validation through confocal and scanning electron microscopy. Their effect on adhesion of pneumococcus to Detroit562 pharyngeal epithelial cells and cytotoxicity was also assessed. Treatment with up to 2% wt/v galactose fluorinated at the C-3 (Gal-3F) and C-6 (Gal-6F) positions had no effect on final planktonic growth after 24 h within glucose-rich BHI medium; however, a reduction in mid-exponential growth rate was observed with 2% Gal-3F. Correlating with the transition towards biofilm formation involving a switch to galactose metabolism, 1–2% wt/v Gal-3F successfully inhibited biofilm formation for four out of five strains tested; however, no activity was observed against 48-h established biofilms, and there was no inhibition of epithelial adhesion. The equivalent concentrations of Gal-6F had no effect on pneumococcal biofilms, suggesting that the site of fluorination is important in activity. The inhibition of biofilm formation by Gal-3F confirms that targeting galactose metabolism is a potentially viable strategy for treating pneumococcal infection and that the rational design of fluorinated glycomimetics offers an alternative approach to targeting pneumococcal biofilms.

## Data Summary

The authors confirm that all supporting data and protocols have been provided within the article or through supplementary data files. File S1 (available in the online Supplementary Material) contains data for all planktonic, biofilm biomass, biofilm viability, epithelial adhesion and cytotoxicity data.

## Introduction

*Streptococcus pneumoniae* (pneumococcus) is a major cause of morbidity and mortality worldwide. The Global Burden of Disease Study 2021 indicates that 505,268 deaths were attributed to lower respiratory tract infection [1] and pneumococcus also causes much sepsis and meningitis. The majority of these deaths occur in developing countries, with the very young (<5 years; 139,267 deaths) and elderly (+70 years; 218,540 deaths) at particular risk [1]. Pneumococcus, an asymptomatic colonizer of the human nasopharynx, is also a leading cause of less severe localized infections such as otitis media, a common infection in young children and a leading cause for antibiotic prescription despite concerns about the development of antimicrobial resistance (AMR) [2, 3].

The transition from asymptomatic colonization to invasive disease is poorly understood. However, it is widely accepted that biofilms, where individual bacteria form aggregates surrounded by a self-produced extracellular matrix, play a prominent role in pneumococcal colonization [4]. Pneumococcal biofilms have been identified *in situ* on adenoid mucosal epithelia from children undergoing adenoidectomy for the treatment of chronic otitis media (COM) and obstructive sleep apnoea [5] and in middle ear biopsy specimens in children with COM [6]. Biofilms are widely acknowledged as a significant problem in clinical settings, primarily through increased antibiotic tolerance compared to their planktonic counterparts, but also due to their propensity to develop AMR through horizontal gene transfer [7]. There is therefore an unmet need for the development of new treatments for pneumococcal biofilm-associated diseases, emphasized by the inclusion of pneumococcus in the WHO priority pathogens list (2024).

Carbohydrate metabolism is of significant importance in pneumococcal pathogenesis [8]. Thirty percent of all transporters encoded by pneumococcus are involved in carbohydrate transport, more than any other bacterial species present in the same niche, reflecting its ability to survive in the nasopharynx where there is a paucity of freely available carbohydrates [9, 10]. To liberate carbohydrates critical for its survival, pneumococcus expresses glycosidases that break down glycoconjugates encountered in the nasopharynx, including mucin, immune cells and epithelial cell surfaces [10]. Evidence suggests that galactose, the predominant carbohydrate in the respiratory tract, is critical for pneumococcal survival and virulence [11, 12]. The cleavage of terminal sialic acid residues from glycoproteins on the epithelial cell surface exposes galactose, facilitating both surface attachment and supporting its metabolism [12, 13]. Pneumococcus also possesses two conserved pathways dedicated to galactose metabolism: the Leloir pathway that metabolizes free environmental galactose into glucose-1-phosphate and the tagatose-6-phosphate pathway that processes galactose attached to other molecules [14]. The tagatose-6-phosphate pathway in particular plays an important role in the early infection process, with an increase in expression of three enzymes in the pathway (LacA, LacB and LacD) being observed during biofilm formation [15]. Pneumococcal *nanA* mutants lacking the capacity to cleave epithelial cell sialic acid residues are also unable to form biofilms *in vivo* [12], whilst decreased lung epithelial cell adherence was observed in mutants lacking a galactose-binding protein (*pblB*) [13], and *lacD* mutants have been shown to have reduced nasopharyngeal colonization in mice [11].

On this evidence, pneumococcal galactose utilization represents an attractive target for the development of novel therapeutic strategies. One potential solution is through the use of fluorinated carbohydrates which have already been utilized for a diverse range of chemical and biological applications [16]. This includes their use as a potential diagnostic tool, with fluorine-18-fluorodeoxyglucose enabling imaging of different bacterial species, including *S. pneumoniae*, during infection using positron emission tomography [17]. Fluorinated carbohydrates have also been applied in the production of glycomimetics derived from *S. pneumoniae* capsular polysaccharides which can be used as potential glycoconjugate vaccines owing to their improved immunogenicity and bioavailability [18]. They have also been used in mechanistic studies, with UDP-2-deoxy-2-fluorogalactose being used to investigate the catalytic mechanism of galactosyltransferase (LgtC) from *Neisseria meningitidis* [19]. Very little research has been undertaken investigating the effect of fluorinated carbohydrates on bacterial biofilms; however, fluorination of 2,4-diacetamide-2,4,6-trideoxygalactose has been shown to act as a metabolic inhibitor of *Helicobacter pylori* glycoprotein synthesis, resulting in reduced biofilm formation [20], whilst the fluorinated pyrroloquinazolinediamine derivative fluorofolin demonstrates antibacterial activity against *Pseudomonas aeruginosa* through its activity as a dihydrofolate reductase inhibitor [21]. In this preliminary study, we investigate the potential of fluorinated galactose analogues as a strategy for preventing and treating pneumococcal biofilms. We hypothesized that the chemical changes to galactose through fluorination would interfere with enzymes involved in the Leloir and tagatose-6-phosphate pathways of pneumococcus, thereby impacting growth and biofilm formation. We specifically investigated galactose fluorinated at the C-3 position (Gal-3F) as this has the potential to interfere with enzymes of the Leloir pathway which act exclusively on carbons 1–4 of galactose.

## Methods

### Bacterial strains and growth conditions

*S. pneumoniae* laboratory strain D39 (serotype 2) and clinical isolates belonging to serotypes 4 (TIGR4), 3 (ST180), 6B (ST138) and 19F (ST179) were selected for assessment of the galactose analogues Gal-3F and Gal-6F, with D39 also being used for the assessment of the glucose analogue Glu-3F. The strains were subcultured from frozen stocks onto brain heart infusion (BHI) agar plates containing 5% defibrinated horse blood (Oxoid, UK) and incubated for 16 h at 37 °C with 5% CO_2_. All growth present on the agar plates was resuspended in BHI broth (Oxoid, UK) and then further diluted to produce ~1×10^5^ c.f.u. ml^−1^ inocula in either BHI (planktonic assays) or 1/5^th^ strength BHI (biofilm assays).

### Planktonic growth and biofilm formation experiments

Flat-bottom 96-well culture plates were inoculated with ~1×10^5^ c.f.u. ml^−1^ per well. d-(+)-Galactose (Thermo Scientific Chemicals, USA), 3-deoxy-3-fluoro-d-galactose (Gal-3F; Biosynth, Switzerland) and 6-deoxy-6-fluoro-d-galactose (Gal-6F; Biosynth, Switzerland) were prepared in BHI broth and added to wells at final concentrations of 0.125, 0.25, 0.5, 1 and 2% wt/v, with BHI used as a control. d-(+)-Glucose (Thermo Scientific Chemicals, USA) and 3-deoxy-3-fluoro-d-glucose (Glu-3F; Biosynth, Switzerland) were prepared in BHI broth and added to wells at a final concentration of 2% wt/v. Cultures were incubated for 24 h at 37 °C with 5% CO_2_ under static conditions, and absorbance (OD_600_) at 24 h was measured using a SpectraMax Plus 34 spectrophotometer (Molecular Devices, UK) to assess planktonic growth (*n*=6). Continuous growth curve measurements for *S. pneumoniae* D39 were performed using a Cerillo Stratus plate reader (Cerillo, USA), with absorbance readings (OD_600_) taken every 3 min for 12 h. For assessment of biofilm formation, spent media were removed, and the biofilms were stained with 0.1% crystal violet (CV) solution for 10 min and then washed three times with Hanks’ Balanced Salt Solution (HBSS; Gibco, USA). The stained biofilms were air-dried, the CV was solubilized using 30% acetic acid and the absorbance (OD_600_) was measured using a SpectraMax Plus 34 spectrophotometer (Molecular Devices, UK) (*n*=6).

### Established biofilm experiments

To produce established biofilms, 12-well culture plates were inoculated with ~1×10^5^ c.f.u. ml^−1^ *S*. *pneumoniae* D39 prepared in 1/5^th^ BHI and grown for 48 h at 37 °C with 5% CO_2_ under static conditions. Half of the spent media were removed and replaced with fresh 1/5^th^ strength BHI at 24 h. Established biofilms were treated with 2% wt/v galactose, Gal-3F or Gal-6F (prepared in HBSS) for 2 h at 37 °C with 5% CO_2_, with HBSS being used as a control. Spent media were removed, and the biofilms were washed three times with HBSS and then resuspended in 1 ml HBSS through cell scraping. Tenfold serial dilutions (NEAT to 10^−7^) were spot-plated in triplicate onto BHI agar plates containing 5% defibrinated horse blood and incubated for 16 h at 37 °C with 5% CO_2_, and viability was assessed through c.f.u. enumeration (*n*=6).

### Confocal laser scanning microscopy

Twenty-four-well optical imaging plates (Azenta Life Sciences, USA) were coated for 1 h in 50 µg ml^−1^ poly-d-lysine (prepared in HBSS), washed twice with HBSS and then inoculated with ~1×10^5^ c.f.u. ml^−1^ *S*. *pneumoniae* D39 prepared in BHI. Galactose, Gal-3F and Gal-6F (prepared in BHI) were added to wells at a final concentration of 2% wt/v, with BHI used as a control. Cultures were incubated for 24 h at 37 °C with 5% CO_2_ under static conditions. Treatments were removed, and the biofilms were stained with live/dead BacLight bacterial viability kit (Molecular Probes, USA) as per the manufacturer’s instructions, washed three times with HBSS and then covered in 60% glycerol (prepared in dH_2_O). Biofilms were imaged using an inverted Leica SP8 confocal laser scanning microscope (Leica Microsystems, UK) with a 63× oil immersion lens and sequential scanning of 1 µm sections.

### Scanning electron microscopy

Twelve-well culture plates containing ethanol-sterilized 13 mm glass coverslips (VWR, UK) were inoculated with ~1×10^5^ c.f.u. ml^−1^ *S*. *pneumoniae* D39 prepared in BHI. Galactose and Gal-3F (prepared in BHI) were added to wells at a final concentration of 2% wt/v, with BHI used as a control. Cultures were incubated for 24 h at 37 °C with 5% CO_2_ under static conditions. Biofilms were washed twice with HBSS and then fixed in 3% glutaraldehyde, 0.1 M sodium cacodylate (pH 7.0) and 0.15% Alcian blue for 2 h at 4 °C. The fixative solution was removed and replaced with 0.1 M sodium cacodylate (pH 7.0) for 1 h; then, the coverslip was placed through an ethanol series (50, 60, 70, 90 and 100%) for 10 min each. The biofilms were critical point dried, sputter coated and then imaged using a ZEISS EVO LS15 scanning electron microscope (ZEISS, Germany).

### Epithelial adhesion and viability

Detroit 562 human pharynx epithelial cells (Public Health England, UK) were grown to confluence in 12-well culture plates coated with 300 µg ml^−1^ rat tail collagen (Gibco, USA). Cells were grown at 37 °C with 5% CO_2_ using Dulbecco’s Modified Eagle Medium (DMEM) containing 4.5 g l^−1^ d-glucose, l-glutamine and sodium pyruvate (Gibco, USA) supplemented with 100 U ml^−1^ penicillin/streptomycin. To assess cytotoxicity, the cells were treated with 2% wt/v galactose and Gal-3F (prepared in DMEM) for 2 h at 37 °C with 5% CO_2_, with DMEM as a control. Treatments were removed, the epithelial cells were dissociated from the surface using 0.25% trypsin-EDTA and centrifuged at 150 ***g*** for 5 min, and the pellet was resuspended in 100 µl DMEM. Cells were stained with 0.2% trypan blue and viability assessed using a Countess II cell counter (Invitrogen, USA) (*n*=4). To assess the effect on pneumococcal adhesion to epithelial cells, it was first confirmed that 2% Gal-3F had no impact on the mid-exponential growth of *S. pneumoniae* D39. To this purpose, *S. pneumoniae* D39 was grown in BHI at 37 °C with 5% CO_2_, with 2% wt/v Gal-3F (prepared in BHI) introduced at 6 h, and absorbance (OD_600_) was measured using a SpectraMax Plus 34 spectrophotometer (Molecular Devices, UK) for 24 h. To assess adhesion, *S. pneumoniae* D39 was grown to ~1×10^6^ c.f.u. ml^−1^ in BHI at 37 °C with 5% CO_2_, centrifuged at 2,500 ***g*** for 5 min, and the pellet was resuspended in either 2% galactose or Gal-3F (prepared in antibiotic-free DMEM). Antibiotic-containing DMEM was removed from confluent Detroit 562 cells in a 12-well plate and washed twice with HBSS, and then, 500 µl of *S. pneumoniae* in the treatments was added to each well for 1 h at 37 °C with 5% CO_2_ [22]. Treatments were then removed, and the cells were washed twice with HBSS and then scraped into 200 µl dH_2_O. Tenfold serial dilutions (NEAT to 10^−5^) were spot-plated in triplicate onto BHI agar plates containing 5% defibrinated horse blood and incubated for 16 h at 37 °C with 5% CO_2_, and viability was assessed through c.f.u. enumeration (*n*=4).

### Statistical analysis

Statistical analysis of the *S. pneumoniae* D39 planktonic and biofilm formation assays was performed using one-way ANOVA and Kruskal–Wallis multiple comparison test. Analyses of the clinical isolate planktonic and biofilm formation data, as well as Detroit 562 epithelial data, were performed using the Mann–Whitney t-test. Comparative data reported with a *P*-value of <0.05 were considered statistically significant.

## Results

### Fluorination of galactose at the C-3 position reduces pneumococcal biofilm formation

Fluorinated galactose analogue concentrations between 0.125 and 2% wt/v were tested based on previous research investigating the effect of different carbohydrate sources on pneumococcal biofilm formation [12]. Deoxyfluorination (where the OH group is replaced by fluorine) at the C-3 and C-6 positions (Fig. 1a) had no effect on planktonic growth of *S. pneumoniae* D39 at 24 h with only a small but insignificant reduction observed at 1–2% wt/v with Gal-3F, whereas the addition of 2% wt/v galactose caused a significant increase in growth (*P*=0.021; Fig. 1b). Continuous measurement of growth, however, indicates that 2% Gal-3F elicits a reduction in exponential growth rate to 0.57 OD_600_ h^−1^ in comparison to the untreated control (1.05 OD_600_ h^−1^). When assessing the impact on biofilm formation, treatment with Gal-3F caused a significant reduction in biomass at 1% wt/v (*P*=0.011) and 2% wt/v (*P*=0.0002), whilst 2% Gal-6F produced a notable but non-significant reduction (Fig. 1c). Confocal imaging revealed that 2% Gal-3F disrupted biofilm formation with reduced surface coverage observed (Fig. 1d), with scanning electron microscopy (SEM) indicating that it also compromises cellular structure (Fig. 1e).

**Fig. 1.**
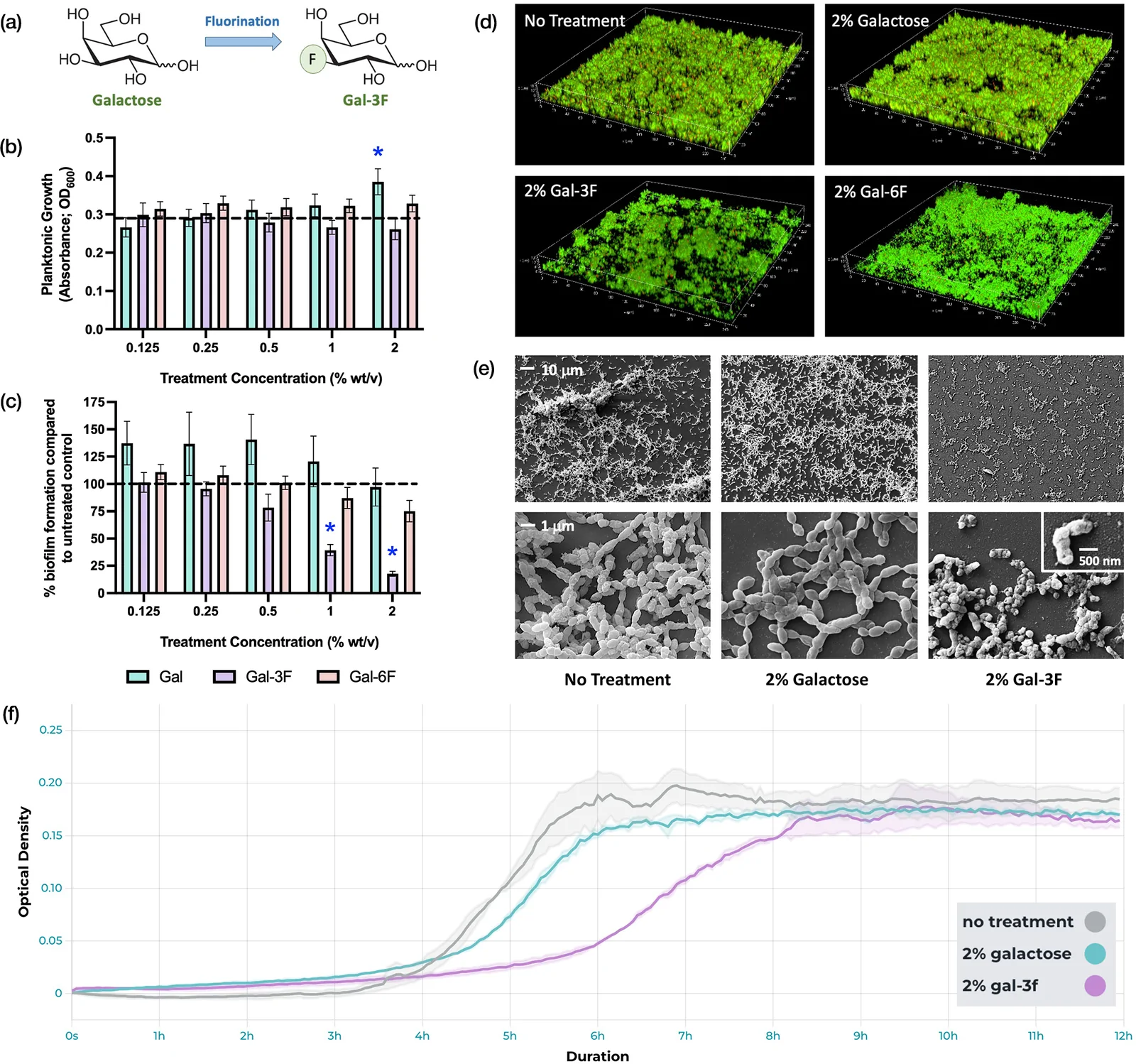
Gal-3F reduces *S. pneumoniae* D39 biofilm formation. (**a**) Structure of galactose and its fluorinated derivative Gal-3F. *S. pneumoniae* D39 was grown in the presence of different concentrations of galactose, Gal-3F and Gal-6F for 24 h. (**b**) Measurement of planktonic growth at 24 h (absorbance; OD_600_) indicates that Gal-3F and Gal-6F had no effect on the final level of growth after 24 h; however, (**f**) continuous measurement of growth indicates that Gal-3F elicits a reduction in exponential growth rate. (**c**) Gal-3F was shown to reduce biofilm formation at 1–2% wt/v concentrations, whereas Gal-6F had no significant effect (measured by CV assay; OD_600_). Gal-3F was shown to impact (**d**) biofilm structure through confocal microscopy using live/dead staining at 63× magnification (green – live, red – dead) and (**e**) cellular structure through SEM. Error bars indicate standard error of the mean (*n*=6); *≤0.05.

### Gal-3F reduces biofilm formation of clinical pneumococcal isolates

Having established that 2% wt/v Gal-3F reduces biofilm formation by the laboratory strain D39, it was decided to ascertain whether its effect was consistent across clinical isolates belonging to different serotypes. Similar to D39, the addition of 2% wt/v galactose significantly increased planktonic growth for strains belonging to serotypes 3, 4, 6B and 19F (Fig. 2a) and was also associated with an increase in biofilm formation (Fig. 2b). When treating with 2% wt/v Gal-3F, no reduction in planktonic growth was observed for TIGR4 or the serotype 6B strain; however, a significant reduction in the growth of the serotype 3 and 19F strains was observed. A significant reduction in biofilm formation was also observed for the TIGR4 (*P*=0.041), serotype 6B (*P*=0.002) and serotype 19F (*P*=0.026) strains (Fig. 2b).

**Fig. 2.**
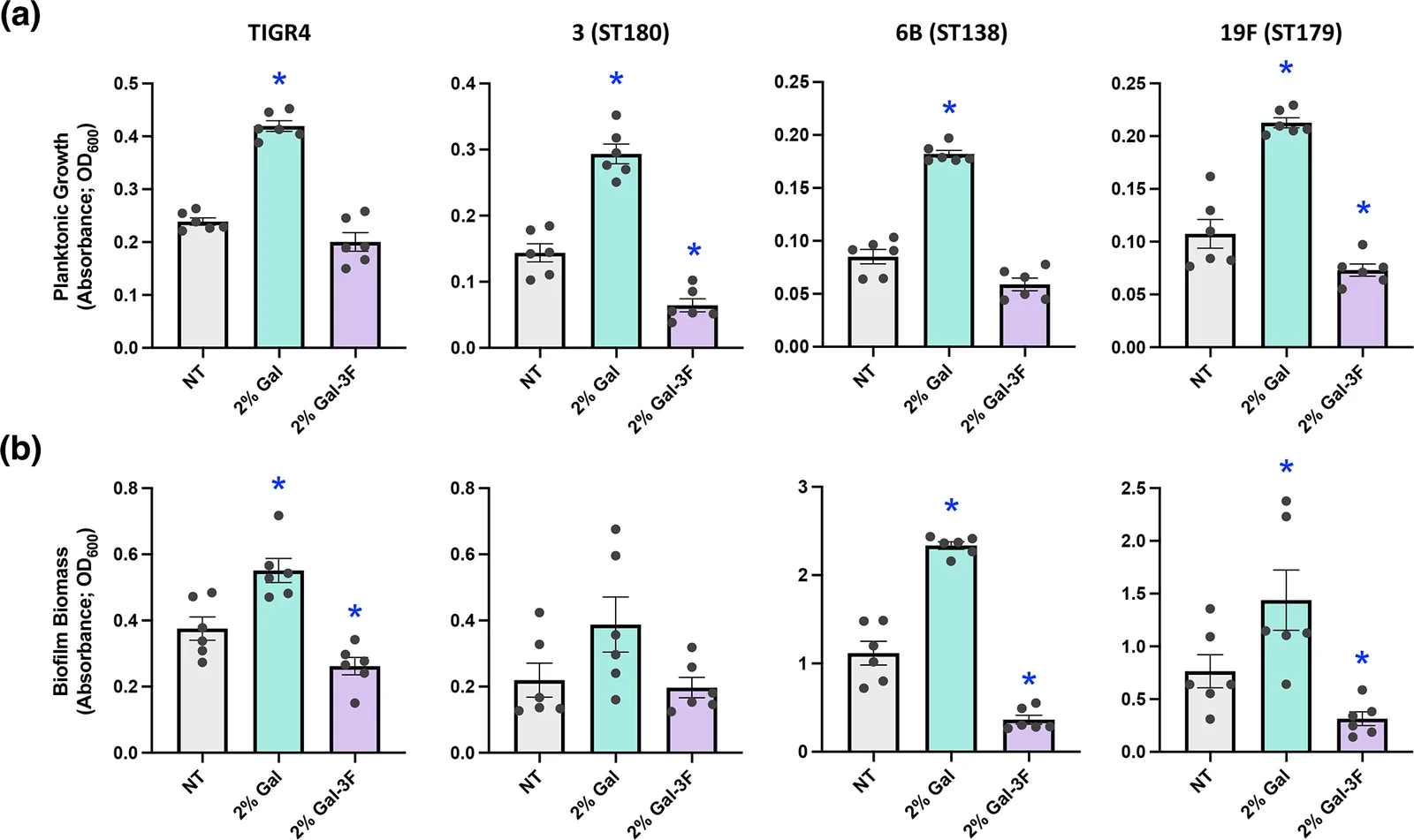
Gal-3F reduces biofilm formation of pneumococcal strains belonging to different serotypes. The effects of 2% wt/v galactose and Gal-3F on (**a**) planktonic growth and (**b**) biofilm formation at 24 h were assessed in four further pneumococcal serotype strains (TIGR4, 3, 6B and 19F). Galactose (2% wt/v) increased planktonic growth and biofilm formation of all strains, whilst Gal-3F (2% wt/v) reduced planktonic growth of two strains (3 and 19F) and biofilm formation for three strains (TIGR4, 6B and 19F). Error bars indicate standard error of the mean (*n*=6); *≤0.05.

### Gal-3F has no effect on established pneumococcal biofilm viability, whereas glucose fluorinated at the C-3 position inhibits planktonic growth

Having confirmed that 2% wt/v Gal-3F reduces biofilm formation across strains from different serotypes, its effect was then assessed against 48-h established *S. pneumoniae* D39 biofilms. Treatment of established biofilms with 2% wt/v galactose, Gal-3F and Gal-6F for 2 h had no effect on biofilm viability, suggesting it only interferes with the process of biofilm formation (Fig. 3a). Knowing that glucose is utilized as the predominant carbon source during pneumococcal planktonic growth, it was also decided to test the effect of glucose fluorinated at the C-3 position (Glu-3F), hypothesizing that it would interfere with planktonic growth. As anticipated, 2% wt/v Glu-3F completely inhibited planktonic growth (*P*=0.002; Fig. 3b) and subsequent biofilm formation (*P*=0.026; Fig. 3c).

**Fig. 3.**
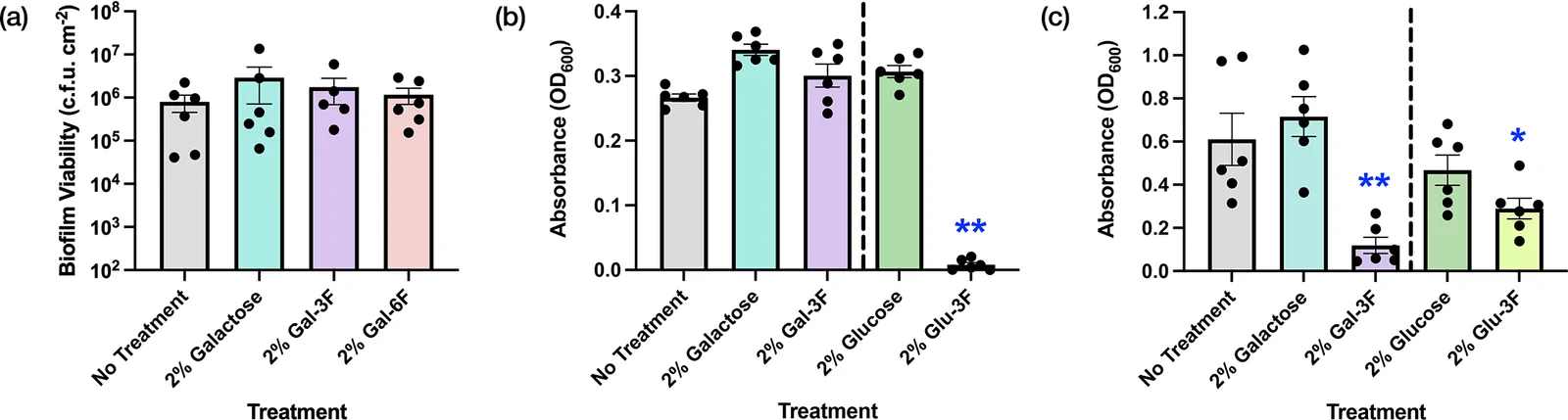
Gal-3F has no effect on established *S. pneumoniae* D39 biofilms, whereas Glu-3F inhibits pneumococcal planktonic growth. (**a**) Established 48-h *S*. *pneumoniae* D39 biofilms were treated with 2% galactose, Gal-3F or Gal-6F for 2 h, and viability was assessed through c.f.u. enumeration, revealing that neither Gal-3F nor Gal-6F had any impact on viability. (**b**) The effect of 2% glucose and Glu-3F on planktonic growth of *S. pneumoniae* D39 was assessed by measuring the change in absorbance (OD_600_) over 24 h, revealing that 2% Glu-3F completely inhibited pneumococcal growth. (**c**) The effect of 2% Glu-3F on biofilm formation was assessed by CV staining (absorbance; OD_600_), revealing a reduction in biofilm formation. Error bars indicate standard error of the mean (*n*=6); *≤0.05, **≤0.01.

### Gal-3F has no impact on initial adhesion of pneumococcus to epithelial cells

Neuraminidase A (NanA) is produced by pneumococcus to cleave glycoproteins on the surface of epithelial cells and expose galactose, thereby supporting host colonization and subsequent metabolism [12]. It was therefore hypothesized that the fluorinated analogue Gal-3F may interfere with host cell adherence. Having confirmed that 2% wt/v Gal-3F has no effect on mid-exponential planktonic growth of *S. pneumoniae* D39 over a 2-h contact period (Fig. 4a), Detroit 562 human pharynx epithelial cells were infected with pneumococcus in the presence of Gal-3F for 1 h, with results indicating that there was no change in initial adhesion (Fig. 4b). The cytotoxic profile of 2% wt/v Gal-3F was also assessed, with 2-h exposure showing no signs of toxicity (Fig. 4c).

**Fig. 4.**
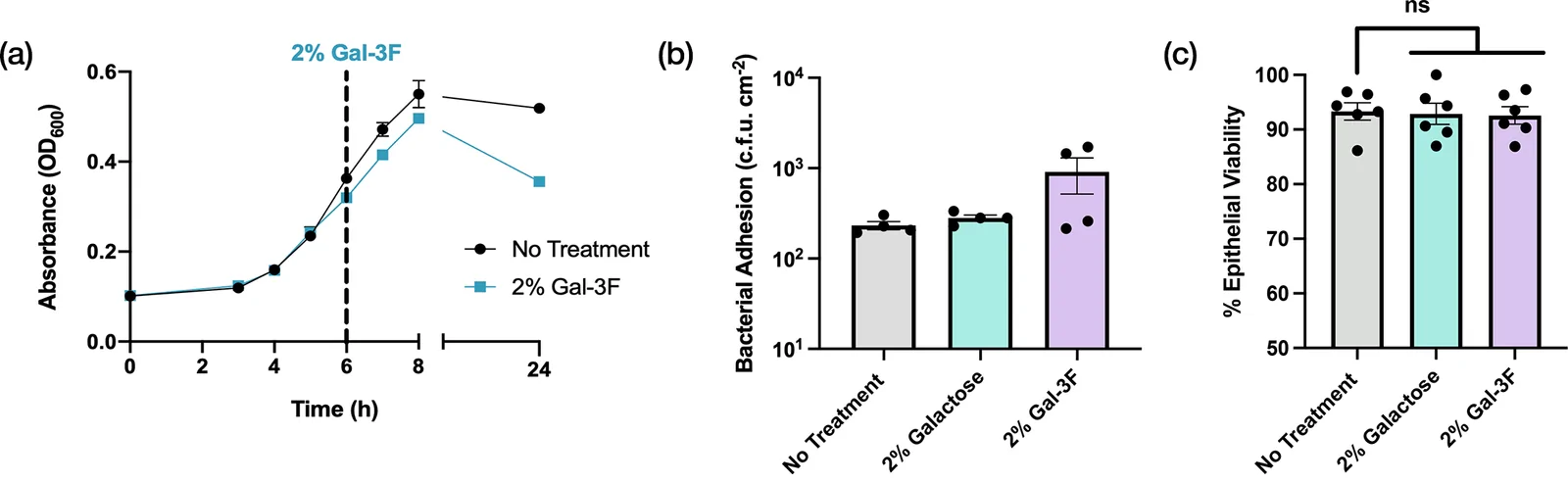
(**a**) Introduction of 2% wt/v Gal-3F to *S. pneumoniae* D39 during mid-exponential growth had no immediate impact on growth rate over 2 h, with the level of growth only being reduced at 24 h. (**b**) Infection of Detroit 562 nasopharyngeal epithelial cells for 1 h with mid-exponential pneumococcus in the presence of 2% Gal-3F demonstrated no impact on immediate cellular adhesion (*n*=4), whilst (**c**) treatment of Detroit 562 cells with 2% Gal-3F for 2 h, absent pneumococcal infection, had no cytotoxic effect (*n*=6). Error bars indicate standard error of the mean; ns, no significance.

## Discussion

Galactose metabolism plays a critical role in *S. pneumoniae* colonization and survival in the human respiratory tract [11–13, 15] and therefore represents an attractive target for the development of new, low-cost therapeutics to reduce the burden on conventional antibiotic use for pneumococcal infection. The potential of carbohydrate-based treatments has been evidenced in a clinical trial involving cystic fibrosis patients with chronic *P. aeruginosa* infection where the inhalation of fucose and galactose, either alone or in combination with antibiotics, reduced the presence of *P. aeruginosa* without any serious side effects [23]; however, it has never been explored for pneumococcus.

In this study, we investigated the potential of commercially available galactose analogues which were fluorinated at the C-3 (Gal-3F) and C-6 sites (Gal-6F), hypothesizing that the structural changes would interfere with their metabolic utilization and subsequently impact biofilm formation. With glycolysis being the primary pathway for generating ATP during planktonic growth, it was anticipated that neither analogue would have an effect on this growth phase. However, despite there being no significant change in growth after 24 h (Fig. 1b), a reduction in exponential growth rate of D39 was observed with 2% wt/v Gal-3F which suggests some level of interference with planktonic metabolic activity. By comparison, complete inhibition was observed when treating planktonic pneumococcus with glucose fluorinated at the C-3 site (Glu-3F; Fig. 3b), confirming interference with glucose metabolism during this phase of growth where it is utilized as the primary carbohydrate source. Glu-3F has previously been investigated in *Escherichia coli* where it had no effect on glucose utilization but instead inhibited the metabolism of other carbohydrates, with enzyme II of the phosphoenolpyruvate-dependent phosphotransferase system being identified as the target [24]. Interestingly, 2-deoxy-d-glucose has also been shown to inhibit biofilm growth in *E. coli* due to a lack of metabolic breakdown which results in the build-up of phosphorylated derivatives that cause growth stress and reduce glucose/mannose uptake [25]. Importantly, 2-deoxy-d-glucose has also been investigated as a potential cancer treatment and shows no cytotoxicity [26], further highlighting the potential of carbohydrate analogues as viable targeted treatments.

Whilst Gal-3F had no effect on D39 planktonic growth after 24 h, a significant reduction in biofilm formation was observed when treating with 1 and 2% wt/v Gal-3F (Fig. 1c–e), suggesting that the analogue was effective during the switch towards galactose metabolism, the primary carbohydrate utilized during biofilm formation [15]. Morphological changes in cellular structure, with most cells appearing stressed and smaller in size (<1 µm), suggest that Gal-3F is compromising cellular integrity which could contribute towards reduced biofilm formation and viability (Fig. 1e). Notably, 2% wt/v Gal-6F had only a small, non-significant effect on biofilm formation (Fig. 1c, d), suggesting that the site of fluorination is important and analogues fluorinated at alternative or multiple sites should be explored in future studies. Notwithstanding, the effect of 2% wt/v Gal-3F was validated using different serotype strains, with three out of four (TIGR4, 6B, 19F) demonstrating significant reduction in biofilm formation (Fig. 2b); however, it should be noted that there was also a small but significant reduction in planktonic growth of strains 3 and 19F (Fig. 2a), confirming the potential to affect this phase of growth. In contrast to this, the provision of the equivalent 2% wt/v galactose caused a significant increase in planktonic growth of all four strains and biofilm formation of three strains (TIGR4, 6B and 19F), suggesting that pneumococcal metabolism had shifted towards galactose as the predominant carbohydrate source in the growth medium, providing further evidence that fluorination impacts its metabolism.

Although Gal-3F inhibits the process of biofilm formation, it demonstrated no effect on the viability of 48-h established biofilms (Fig. 3a). Pneumococcus possesses two galactose metabolic pathways, the Leloir and tagatose-6-phosphate pathways, with only the enzymes of the tagatose-6-phosphate pathway shown to be increased in expression in pneumococcal biofilms [15]. The lack of effect on established biofilms therefore suggests that the Leloir pathway might be the primary target, with a recent study showing that Gal-3F completely prevented induction of galactokinase (GalK) (Leloir pathway) whilst only reducing the induction of LacA (tagatose-6-phosphate pathway) [14]. The Leloir pathway acts almost exclusively on carbons 1–4 of galactose, meaning that fluorination at C-6 (Gal-6F) will have little-to-no effect on its metabolism, potentially explaining why there was only a minor reduction in biofilm formation. Gal-3F, however, can be phosphorylated by the second enzyme in the pathway, GalK, to produce 3-deoxy-3-fluorogalactose-1-phosphate, which acts as a competitive inhibitor for the third enzyme in the pathway, galactose-1-phosphate uridylyltransferase (GalT). Inhibition of GalT can result in the toxic accumulation of phosphorylated galactose which has the capacity to induce cellular stress, a phenomenon previously evidenced in other bacterial species and yeast [25, 27], and is evidenced in this study by the change in cellular morphology and reduced biofilm formation. It would also make sense to design analogues with the potential to interfere with the tagatose-6-phosphate pathway, for the purpose of not only targeting already established biofilms but also circumnavigating any detrimental effects on the Leloir pathway in human cells (the tagatose-6-phosphate pathway is not found in humans). For instance, 2-fluoro-2-deoxygalactose-1-phosphate has been shown to inhibit galactose-1-phosphate in the Leloir pathway of human embryonic kidney (HEK-293) cells [28], highlighting the importance of rational design. In addition to investigating the effect of Gal-3F on biofilm formation, we also assessed its impact on pneumococcal attachment to Detroit 562 nasopharyngeal epithelial cells, hypothesizing that it would competitively inhibit adhesion to galactose exposed by NanA [12]. No evidence of epithelial adhesion was observed; however, we confirmed that exposure to 2% wt/v Gal-3F for 2 h had no cytotoxic effects (Fig. 4). Whilst further investigation into the use of fluorinated galactose analogues and their mechanism of action is required, this preliminary research highlights their potential as a targeted approach for treating pneumococcal biofilm-associated infections.

## Supplementary material

10.1099/acmi.0.001196.v4 Supplementary Material 1.

## Abbreviations

AMR: antimicrobial resistance
BHI: brain heart infusion
COM: chronic otitis media
CV: crystal violet
DMEM: Dulbecco’s Modified Eagle Medium
GalK: galactokinase
GalT: galactose-1-phosphate uridylyltransferase
HBSS: Hanks’ Balanced Salt Solution
NanA: Neuraminidase A
SEM: scanning electron microscopy

## References

[1] Kong Z, Xiong J, Chen L, Peng K, Liu H (2025). The global, regional, and national burden of lower respiratory infections caused by *Streptococcus pneumoniae* Between 1990 and 2021. Healthcare.

[2] Vergison A, Dagan R, Arguedas A, Bonhoeffer J, Cohen R (2010). Otitis media and its consequences: beyond the earache. Lancet Infect Dis.

[3] Boonacker CWB, Rovers MM, Browning GG, Hoes AW, Schilder AGM (2014). Adenoidectomy with or without grommets for children with otitis media: an individual patient data meta-analysis. Health Technol Assess.

[4] Hall-Stoodley L, Nistico L, Sambanthamoorthy K, Dice B, Nguyen D (2008). Characterization of biofilm matrix, degradation by DNase treatment and evidence of capsule downregulation in *Streptococcus pneumoniae* clinical isolates. BMC Microbiol.

[5] Hoa M, Tomovic S, Nistico L, Hall-Stoodley L, Stoodley P (2009). Identification of adenoid biofilms with middle ear pathogens in otitis-prone children utilizing SEM and FISH. Int J Pediatr Otorhinolaryngol.

[6] Hall-Stoodley L, Hu FZ, Gieseke A, Nistico L, Nguyen D (2006). Direct detection of bacterial biofilms on the middle-ear mucosa of children with chronic otitis media. JAMA.

[7] Marks LR, Reddinger RM, Hakansson AP (2012). High levels of genetic recombination during nasopharyngeal carriage and biofilm formation in *Streptococcus pneumoniae*. *mBio*.

[8] Minhas V, Paton JC, Trappetti C (2021). Sickly Sweet - how sugar utilization impacts pneumococcal disease progression. Trends Microbiol.

[9] Burnaugh AM, Frantz LJ, King SJ (2008). Growth of *Streptococcus pneumoniae* on human glycoconjugates is dependent upon the sequential activity of bacterial exoglycosidases. J Bacteriol.

[10] King SJ (2010). Pneumococcal modification of host sugars: a major contributor to colonization of the human airway?. Mol Oral Microbiol.

[11] Paixão L, Oliveira J, Veríssimo A, Vinga S, Lourenço EC (2015). Host glycan sugar-specific pathways in *Streptococcus pneumoniae*: galactose as a key sugar in colonisation and infection [corrected]. PLoS ONE.

[12] Blanchette KA, Shenoy AT, Milner J, Gilley RP, McClure E (2016). Neuraminidase A-exposed galactose promotes *Streptococcus pneumoniae* biofilm formation during colonization. *Infect Immun*.

[13] Hsieh YC, Lin TL, Lin CM, Wang JT (2015). Identification of PblB mediating galactose-specific adhesion in a successful *Streptococcus pneumoniae* clone. Sci Rep.

[14] Kareem BO, Gazioglu O, Mueller Brown K, Habtom M, Glanville DG (2024). Environmental and genetic regulation of *Streptococcus pneumoniae* galactose catabolic pathways. Nat Commun.

[15] Allan RN, Skipp P, Jefferies J, Clarke SC, Faust SN (2014). Pronounced metabolic changes in adaptation to biofilm growth by *Streptococcus pneumoniae*. PLoS One.

[16] Linclau B, Ardá A, Reichardt N-C, Sollogoub M, Unione L (2020). Fluorinated carbohydrates as chemical probes for molecular recognition studies. Current status and perspectives. *Chem Soc Rev*.

[17] Heuker M, Sijbesma JWA, Aguilar Suárez R, de Jong JR, Boersma HH (2017). In vitro imaging of bacteria using 18F-fluorodeoxyglucose micro positron emission tomography. Sci Rep.

[18] Gast D, Neidig S, Reindl M, Hoffmann-Röder A (2025). Synthesis of fluorinated glycotope mimetics derived from *Streptococcus pneumoniae* serotype 8 CPS. Int J Mol Sci.

[19] Persson K, Ly HD, Dieckelmann M, Wakarchuk WW, Withers SG (2001). Crystal structure of the retaining galactosyltransferase LgtC from Neisseria meningitidis in complex with donor and acceptor sugar analogs. Nat Struct Biol.

[20] Williams DA, Pradhan K, Paul A, Olin IR, Tuck OT (2020). Metabolic inhibitors of bacterial glycan biosynthesis. Chem Sci.

[21] Chain C, Sheehan JP, Xu X, Ghaffari S, Godbole A (2024). A folate inhibitor exploits metabolic differences in *Pseudomonas aeruginosa* for narrow-spectrum targeting. Nat Microbiol.

[22] Andersson B, Eriksson B, Falsen E, Fogh A, Hanson LA (1981). Adhesion of Streptococcus pneumoniae to human pharyngeal epithelial cells in vitro: differences in adhesive capacity among strains isolated from subjects with otitis media, septicemia, or meningitis or from healthy carriers. Infect Immun.

[23] Hauber HP, Schulz M, Pforte A, Mack D, Zabel P (2008). Inhalation with fucose and galactose for treatment of *Pseudomonas aeruginosa* in cystic fibrosis patients. Int J Med Sci.

[24] Miles RJ, Pirt SJ (1972). Inhibition of the utilization of lactose and other carbon sources by 3-deoxy-3-fluoro-D-glucose in *Escherichia coli*. Biochem J.

[25] Sutrina SL, Griffith MSJ, Lafeuillee C (2016). 2-Deoxy-d-glucose is a potent inhibitor of biofilm growth in *Escherichia coli*. *Microbiology (Reading*).

[26] Leung HJ, Duran EM, Kurtoglu M, Andreansky S, Lampidis TJ (2012). Activation of the unfolded protein response by 2-deoxy-D-glucose inhibits Kaposi’s sarcoma-associated herpesvirus replication and gene expression. Antimicrob Agents Chemother.

[27] Gibney PA, Schieler A, Chen JC, Bacha-Hummel JM, Botstein M (2018). Common and divergent features of galactose-1-phosphate and fructose-1-phosphate toxicity in yeast. *Mol Biol Cell*.

[28] Janes V, Grabany S, Delbrouck J, Vincent SP, Gottschalk J (2020). Fluorinated galactoses inhibit galactose-1-phosphate uridyltransferase and metabolically induce galactosemia-like phenotypes in HEK-293 Cells. Cells.

